# Poster Session II - A280 MIXED-METHOD QUANTITATIVE SURVEY AND QUALITATIVE PATIENT-LED FOCUS GROUP STUDY EXPLORING PATIENT PERSPECTIVES ON DIGITAL TOOLS FOR INFLAMMATORY BOWEL DISEASE SELF-CARE

**DOI:** 10.1093/jcag/gwaf042.280

**Published:** 2026-02-13

**Authors:** S Y Quan, K Wong, S Zelinsky, J Mikhail, A Pounder, J Cromwell, K D Chappell, M Fox, D Kao, C Seow, L Ajibulu

**Affiliations:** University of Alberta, Edmonton, AB, Canada; University of Alberta, Edmonton, AB, Canada; University of Calgary, Calgary, AB, Canada; University of Alberta, Edmonton, AB, Canada; University of Alberta, Edmonton, AB, Canada; University of Alberta, Edmonton, AB, Canada; University of Alberta, Edmonton, AB, Canada; University of Alberta, Edmonton, AB, Canada; University of Alberta, Edmonton, AB, Canada; University of Calgary, Calgary, AB, Canada; University of Alberta, Edmonton, AB, Canada

## Abstract

**Background:**

Inflammatory Bowel Disease (IBD) is chronic condition requiring a multi-disciplinary, patient-centered approach to address its complex physical, emotional and psychosocial impacts. Digital tools integrated into electronic health records can help promote patient engagement in the management and monitoring of their disease by facilitating communication and information sharing between patients and care providers.

**Aims:**

To explore patient perspectives on the role of digital health tools in the self-management of IBD to guide the development of a more responsive and inclusive bundled digital tool (MyIBDToolkit).

**Methods:**

This study employed a mixed-method quantitative and qualitative approach to gaining insights to patient experiences with digital tools for self-care. Patients were recruited in IBD clinics and endoscopy units in Alberta, Canada between May 2024 to September 2024 using posters with QR codes linked to a digital consent and survey. The subgroup of patients who indicated interest in further participation were enrolled into the focus group study. Four Patient Research Partners co-developed and led five online 2-hour focus group sessions between August to September 2024. Recorded sessions were transcribed using Rev.com. Data was anonymized and analyzed using a deductive thematic approach with NVivo-14 software. A codebook was developed and validated by two researchers independently.

**Results:**

Survey data was obtained from 78 participants (Median age 30 years, 60% female). At the time of the survey, 37 out of 78 participants were currently using digital tools in their IBD management and 28 of the 78 participants reported having never used digital tools previously. Twenty-two survey participants took part in the focus group. There were no significant differences in the focus group subset and larger survey population in terms of age, sex, IBD diagnosis and disease duration. Data analysis revealed 29 themes and 51 sub-themes across three main domains: self-management strategies, digital platform features and content, and barriers to use. Key insights included the importance of tracking diet, stress level, and symptoms, maintaining overall physical health, access to peer support, usability of the digital tool, maintaining online privacy, and engaging in meaningful communication with healthcare providers.

**Conclusions:**

Patient feedback throughout the research process was key in identifying actionable priorities for improving digital health tools to enhance the effectiveness and acceptability of these tools in chronic disease management. These findings will guide development of the MyIBDToolkit.

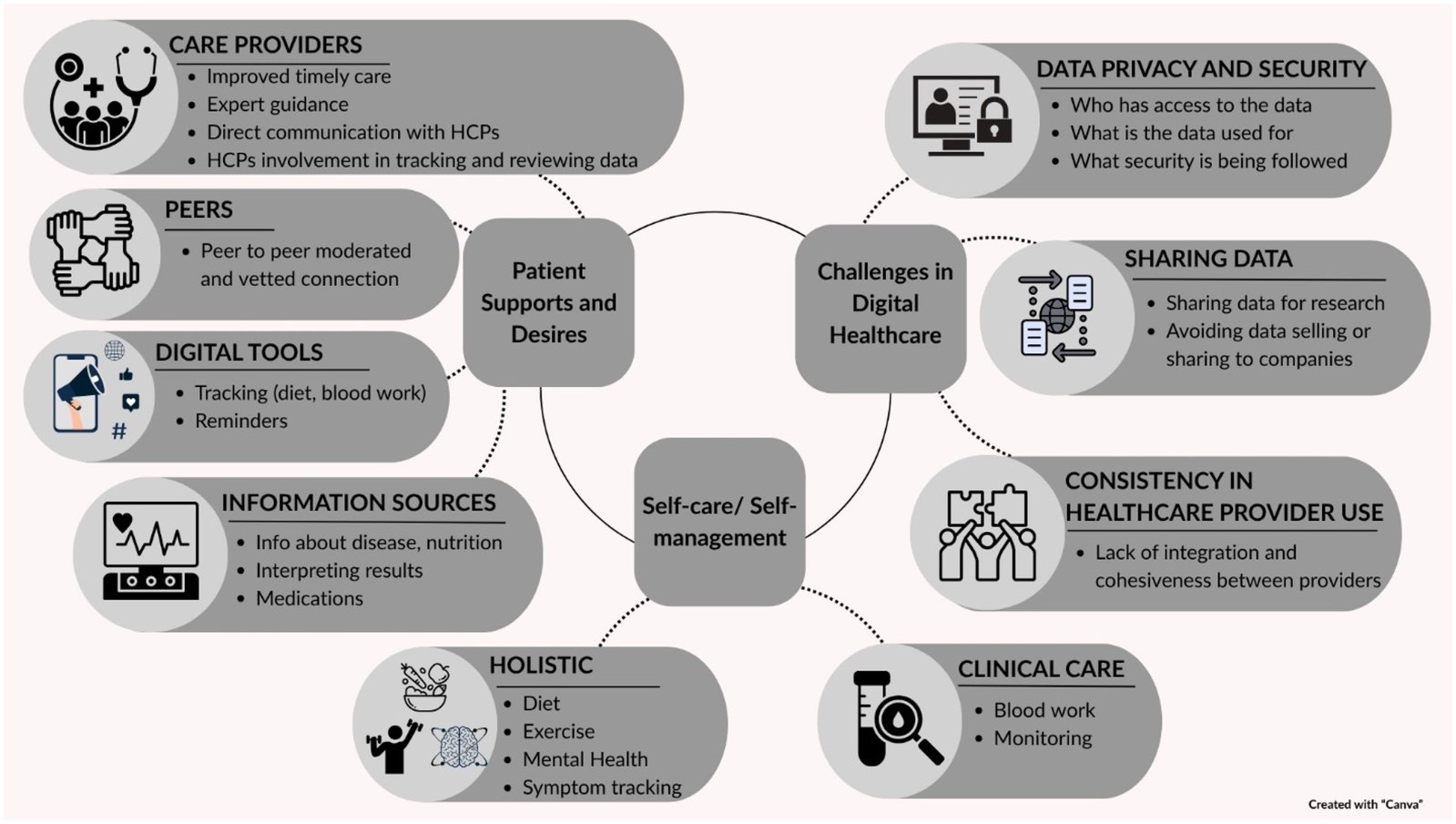

**Funding Agencies:**

Alberta Innovates and AbSPORU Patient Engagement Team

